# A simple and efficient method for the long-term preservation of plant cell suspension cultures

**DOI:** 10.1186/1746-4811-8-4

**Published:** 2012-01-30

**Authors:** Anne-Marie Boisson, Elisabeth Gout, Richard Bligny, Corinne Rivasseau

**Affiliations:** 1 Commissariat à l'Energie Atomique, institut de Recherche en Technologies et Sciences pour le Vivant, Laboratoire de Physiologie Cellulaire Végétale, Unité Mixte de Recherche 5168 CNRS, UJF, INRA, CEA, F-38054 Grenoble, France

**Keywords:** Plant cell suspension, *Acer pseudoplatanus*, *Arabidopsis thaliana*, cell preservation, *in vitro *and *in vivo *NMR spectroscopy, low temperature, phosphate starvation

## Abstract

**Background:**

The repeated weekly subculture of plant cell suspension is labour intensive and increases the risk of variation from parental cells lines. Most of the procedures to preserve cultures are based on controlled freezing/thawing and storage in liquid nitrogen. However, cells viability after unfreezing is uncertain. The long-term storage and regeneration of plant cell cultures remains a priority.

**Results:**

Sycamore (*Acer pseudoplatanus*) and Arabidopsis cell were preserved over six months as suspensions cultures in a phosphate-free nutrient medium at 5°C. The cell recovery monitored via gas exchange measurements and metabolic profiling using *in vitro *and *in vivo *^13^C- and ^31^P-NMR took a couple of hours, and cell growth restarted without appreciable delay. No measurable cell death was observed.

**Conclusion:**

We provide a simple method to preserve physiologically homogenous plant cell cultures without subculture over several months. The protocol based on the blockage of cell growth and low culture temperature is robust for heterotrophic and semi-autotrophic cells and should be adjustable to cell lines other than those utilised in this study. It requires no specialized equipment and is suitable for routine laboratory use.

## Background

Suspension culture of isolated plant cells is an invaluable tool for providing the material for high-throughput studies such as metabolic analyses, production of secondary plant products, and herbicide discovery. It enables easy experimentation on physiologically and biochemically homogenous population of cells. Different methods for cultivating large quantities of plant cells in liquid nutrient medium (NM) have been described for a long time [1-4]. These methods are based on the subculture of cell suspensions having reached their growth plateau when most of the nutrients initially added to NM, particularly carbohydrates, are metabolised. It leads to more or less homogenous cell populations and usually induces a growth delay (lag phase) following subculture [5]. It has been shown that obtaining homogenous cell suspension cultures requires sophisticated apparatus such as chemostats that optimize NM and cell growth [6]. Alternatively, subcultures every one or two days also yields homogenous cell populations [7], but involve much handling and maintenance that is therefore difficult to perform over long periods of time.

For this reason, alternative procedures to preserve newly optimized cell suspension cultures, ideally for indefinite periods, have been proposed. Apart from the maintenance of cell callus on solid media which lead to appreciable delays to initiate homogenous cell suspension cultures, most of the procedures are based on controlled freezing/thawing and storage in liquid nitrogen [8-12]. However, the viability of the cells after unfreezing is generally low and long lag phases before full recovery of cell culture growth are always mentioned by authors. The highest viability (up to 90%) was observed by Menges and Murray [13] after cryopreservation of Arabidopsis and tobacco cells in the presence of DMSO and sorbitol. Nevertheless, even in this case, it takes at least one week for cells to recover normal post-thaw growth and full re-establishment, and there is a risk that preserved cell lines may differ from the original ones [14].

Here, we describe a procedure aiming at preserving higher plant cell populations in their suspension nutrient medium over several months, keeping them homogenous and ready to restart growth after their return to standard culture conditions. The main problem is that, in standard cultures, carbon substrates are consumed within less than two weeks. Afterwards autophagic process and cell death are observed [15,16]. In order to diminish the carbohydrate consumption by cells, cultures were carried out at low temperature in a first series of assays. Cultures were then carried out at low temperature in the absence of phosphate (Pi) so as to induce the arrest of cell growth [17]. In order to validate this procedure, the cell's physiological and biochemical states were monitored both via the measurement of cell growth and gas exchange and by *in vivo *and *in vitro *metabolic profiling utilising ^31^P- and ^13^C-NMR [18-20]. Indeed, NMR monitoring permits the detection of early signals of metabolic disruption related to carbon deprivation [21] leading to the expression of autophagy-related genes [22]. Heterotrophic sycamore (*Acer pseudoplatanus *L.) cells and semi-autotrophic Arabidopsis cells (*Arabidopsis thaliana *L., wild type, Columbia ecotype) were used to compare results obtained with non-chlorophylous cells of cambial origin to those obtained with illuminated chlorophylous cells of leaf parenchyma origin. It was expected that, due to photosynthesis activity, the cellular need for exogenous carbohydrate would be lower, thus permitting a reduction in NM renewal.

## Results and discussion

### Growth of sycamore and Arabidopsis suspension-cultured cells at 22°C and 5°C

**Figure 1:**
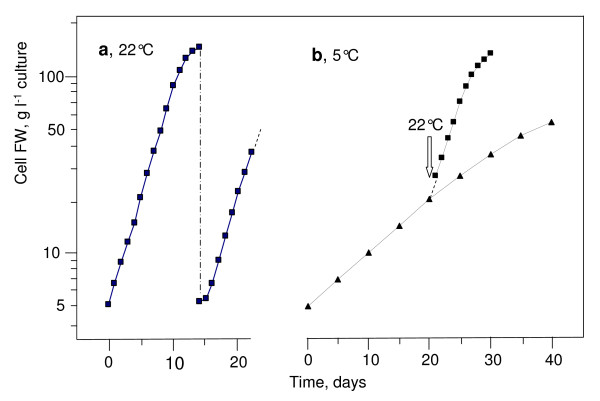
**Growth of suspension-cultured sycamore cells at 22°C and 5°C**. Cells were cultivated in Lamport's nutrient medium as described in "Material and Methods". They originated from an exponentially growing cell suspension maintained at 22°C and repeatedly subcultured before the growth plateau. The cell concentration (FW) at time zero was 5.0 mg ml^-1 ^culture. At 22°C **(a)**, cells grew exponentially for 10 d (linear on semi-logarithmic plot) before reaching a plateau. At day 14, the dilution of cell suspension to their initial concentration (subculture) restarted the exponential cell growth after a 2-d lag. At 5°C **(b)**, cells grew exponentially for 40 d before reaching a plateau. In separate experiments (arrow in Figure 1b), the temperature was set back to 22°C after 20 d of culture at 5°C. This experiment was repeated five times. In semi-logarithmic plot, standard deviation is of the order of the symbol size.

At 22°C, the fresh weight (FW) of weekly subcultured sycamore cells increases exponentially with time, without a lag phase ([7] and Figure 1A). A plateau corresponding to a cell FW of 150 ± 15 g l^-1 ^of culture is reached after two weeks. If cells are subcultured at this stage, a 2-d lag phase attributed to the exhaustion of sucrose supply and to the beginning of the related process of autophagy is observed. At 5°C, the cell density doubling time is much longer: 10 d vs 2.5-2.7 d at 22°C (Figure 1B). Interestingly the pH of their NM, initially adjusted to 5.7, increased progressively up to 7.1-7.3. This is probably due to the presence of nitrate as the only nitrogen source in Lamport's NM and to the lower emission of acidifying CO_2_, whereas it remains below 6.5 at 22°C.

The growth of Arabidopsis cells cultivated under photomixotrophic conditions at 22°C showed a similar profile (Additional File 1A) and a plateau corresponding to a cell concentration of 200 ± 20 mg ml^-1 ^culture was reached within ca 10 days. Here too, lowering the temperature resulted in a marked decrease of cell culture growth rate (Additional File 1B). The cell density doubling time increased from 2.1-2.3 d to ca 8 d. Cultures performed in Murashige and Skoog medium use ammonium and nitrate as a nitrogen source, leading the NM's pH to decrease progressively to ca. 5.0.

The growth rates of both types of cells incubated over one month at 5°C recovered standard values as soon as the culture temperature was set back to 22°C (Figure 1 and Additional File 1 arrows). Despite this extended period of very slow growth, no significant lag phase was observed before cell growth restarted at 22°C, suggesting that the preservation of cells at low temperature did not substantially modify their physiological properties. To confirm this conclusion, measurements of O_2_-uptake by cells and NMR-based control of their metabolite profile were performed.

### O_2_-uptake at 22°C and 5°C

The O_2 _uptake rates of sycamore and Arabidopsis cells cultivated at 22°C, harvested during the exponential phase of growth, and measured at 22°C in the dark, were 350 ± 25 and 480 ± 40 nmol O_2 _min^-1 ^g^-1 ^cell FW, respectively (Table 1). In contrast, the O_2 _uptake rate of the same cells cultivated at 5°C for 20 d, and measured at 5°C in the dark, was much lower, averaging 72 ± 7 and 105 ± 10 nmol O_2 _min^-1 ^g^-1 ^cell FW, respectively. Interestingly, the uncoupled rate of O_2 _consumption measured in the presence of 2 μM FCCP showed a higher relative increase at low temperature. This increase was close to 60% at 22°C and reached 100% at 5°C in sycamore and Arabidopsis cells. This indicates that the decrease of the normal (coupled) cell respiration observed at low temperature was not caused by a limitation of substrate supply to mitochondria or by the intrinsic mitochondria oxidative functioning. It is more likely caused by the general decrease of cell metabolism activity and the correlated decrease of cell need for nucleotide triphosphates (NTP). Increasing the temperature to 22°C after a 20-d incubation at 5°C led to the full recovery of coupled and uncoupled cell respiration within 10 minutes.

**Table 1:** O_2_-uptake by sycamore and Arabidopsis cells incubated at different temperatures

	sycamore		Arabidopsis		
	
Temperature		+ 2 μM FCCP		+ 2 μM FCCP	+ light
22°C	350 ± 25	550 ± 50	480 ± 40	780 ± 70	330 ± 30

5°C	72 ± 6	145 ± 12	105 ± 10	200 ± 15	30 ± 3

5°C→22°C	360 ± 30	560 ± 50	470 ± 40	750 ± 70	290 ± 25

The O_2_-uptake by Sycamore and Arabidopsis cells harvested 5 d after subculture at 22°C and 20 d after subculture at 5°C was measured in their respective culture media at 22°C or 5°C. In addition, the O_2_-uptake by cells cultivated for 20 d at 5°C was measured at 22°C after a 10-min recovery (5°C→22°C). O_2_-uptake rates were measured in the dark, except for Arabidopsis cells when specified (+ light), as indicated in "Material and Methods". They are expressed as nmol O_2 _consumed min^-1 ^g^-1 ^cell FW. Values are means ± SD (*n *= 5). FCCP, cyanide *p*-trifluoromethoxyphenylhydrazone.

The illumination of Arabidopsis cells in the O_2_-electrode chamber decreased the O_2_-uptake by cells by ca 30% at 22°C, probably due to the contribution of O_2_-generating photosynthesis activity (Table 1). Indeed, in the light, these chlorophyllous cells incorporate ^13^CO_2 _into carbon metabolites [unpublished result; 23]. At 5°C, illuminating Arabidopsis cells also decreased the O_2_-uptake (Table 1). In addition, this decrease was proportionally greater than that observed at 22°C suggesting that, at low temperature, the photosynthesis activity of illuminated Arabidopsis cells was affected less than their respiration. Nevertheless, in these semi-autotrophic cells, even at 5°C, the O_2 _produced by photosynthesis never compensated for the O_2 _consumed by respiration.

### Metabolite profile of cells cultivated at 22°C and 5°C

**Figure 2:**
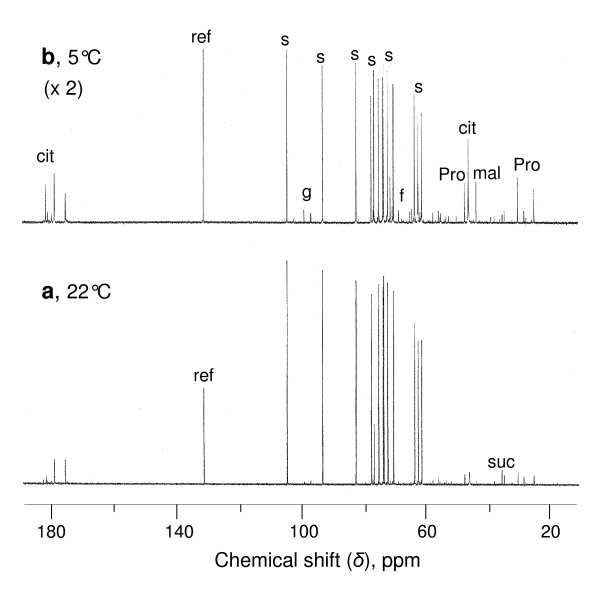
**Proton-decoupled ^13^C-NMR spectra of perchloric acid extracts of sycamore cells grown at 22°C and 5°C**. Cells were harvested 5 d after subculture at 22°C (**a**) and 20 d after subculture at 5°C (**b**). Peak assignments are as follows: ref, reference (maleate) used for quantification; s, sucrose; g, glucose; f, fructose; cit, citrate; mal, malate; suc, succinate; Pro, proline.

The metabolic profiles of sycamore and Arabidopsis cells grown at 22°C and 5°C were analysed from perchloric acid (PCA) extracts using ^13^C- and ^31^P-NMR spectroscopy. ^13^C-NMR spectra show that lowering the temperature in sycamore cells induces significant change in their carbohydrate and organic acid stores (Figure 2). For example, sucrose decreased from 70-80 μmol g^-1 ^cell FW to 30-35 μmol g^-1 ^cell FW, whereas citrate increased from 5.5-6.5 μmol g^-1 ^cell FW to 40-48 μmol g^-1 ^cell FW and proline increased from 2.5-3.5 μmol g^-1 ^cell FW to 8.5-9.5 μmol g^-1 ^cell FW. Arabidopsis cells did not contain ^13^C-NMR-detectable carbohydrates, neither at 22°C nor at 5°C (Additional File 2). This means that their intracellular concentration was below 0.5 μmol g^-1 ^cell FW, whatever the culture temperature. Concerning organic acids, glutamine concentration increased fourfold at low temperature, reaching 25-30 μmol g^-1 ^cell FW, and aspartate twofold, reaching 9-10 μmol g^-1 ^cell FW. On the contrary, citrate and malate, but not fumarate, decreased significantly. The very low level of soluble carbohydrates present in Arabidopsis cells and the low amount of starch [24] could contribute to why a dramatic fall of physiological activity and extensive transcriptomic responses are observed a few hours only after the onset of sugar deprivation leading to cell death within 24 h [25]. Indeed, in suspension-cultured Arabidopsis cells more than one third of the proteins content is degraded during the first 24 h of sucrose starvation [22], whereas it takes 2-3 d in sycamore cells [26,27].

Increasing the temperature to 22°C after a one-month incubation at 5°C led to the recovery of standard ^13^C-NMR profiles for both types of cells within a couple of hours. For example, proline and glutamine which accumulate at low temperature and/or in the absence of Pi [28-30] were metabolised within a few hours. The accumulation of citrate and malate in sycamore cells could be attributed to the fact that the pH of Lamport's NM alkalizes with time [31]. The contrary was observed with Arabidopsis cells grown in Murashige and Skoog's NM which acidifies.

**Figure 3:**
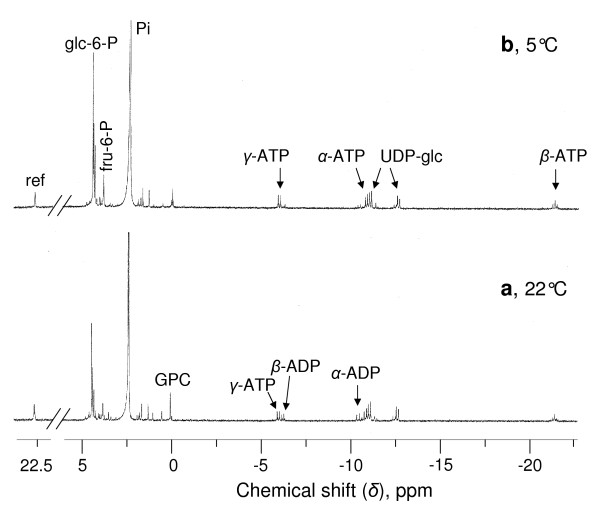
**Proton-decoupled ^31^P-NMR spectra of perchloric acid extracts of sycamore cells grown at 22°C and 5°C**. Cells were harvested 5 d after subculture at 22°C (**a**) and 20 d after subculture at 5°C (**b**). Peak assignments are as follows: ref, reference (methylphosphonate) used for quantification; glc-6-P, glucose 6-phosphate; fru-6-P, fructose 6-P; Pi, inorganic phosphate; GPC, glycerophosphocholine; UDP-glc, uridine-5'-diphosphate-*α*-D-glucose.

^31^P-NMR spectra (Figure 3) indicate that sycamore cells accumulate nearly twice as much hexoses phosphate and nucleotides at 5°C than at 22°C (0.70-0.80 μmol glucose 6-P g^-1 ^cell FW at 5°C vs 0.45-0.50 μmol at 22°C, and 140-160 nmol ATP g^-1 ^cell FW at 5°C vs 80-90 nmol at 22°C). Similar results were obtained with Arabidopsis cells with the exception of glycerophosphoglycerol (GPG) accumulating at low temperature (Additional File 3). GPG is a phosphodiester involved in the metabolism of phosphatidylglycerol, the only abundant phospholipid in chloroplast membranes. Importantly, in both cell lines, neither P-choline nor asparagine accumulated, indicating the absence of cytosplasmic autophagy processes known to occur in cells lacking carbon supply [15,16].

Increasing the temperature to 22°C after 20 days of incubation at 5°C led to the recovery of standard cell hexose-P and nucleotide concentrations within 10-15 minutes.

### Carbohydrate stores

**Figure 4:**
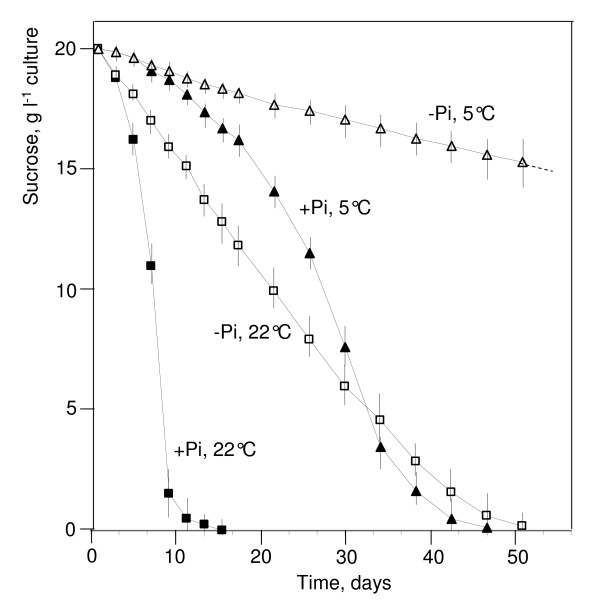
**Temperature and phosphate effect on sucrose decrease in the nutrient medium of sycamore cell culture**. Time course decrease of sucrose was measured in the nutrient medium of sycamore cell cultures at 22°C and 5°C, in the presence or absence of inorganic phosphate (Pi). Cells were initially incubated for 5 d in Pi-supplied or Pi-free NM. The cell concentration (FW) at time zero was 20 mg ml^-1 ^culture in Pi-supplied NM and 30 mg ml^-1 ^culture in Pi-free NM. Sucrose was quantified as described in "Material and Methods". Values are given as means ± SE (n = 3).

The consumption of sucrose in a nutrient medium is proportional to the number of cells growing in this medium and, consequently, it increases exponentially in accordance with the exponential growth of cell populations. At 22°C, the rate of carbohydrate consumption by sycamore cells was 40-50 mg sucrose d^-1 ^g^-1 ^cell FW (Figure 4 and Table 2). It took ca 10 d for the sucrose initially present in the NM, where cells were subcultured, to be exhausted. At 5°C, the rate of sucrose consumption per gram of cell FW was 4-5 times lower (Figure 4), and it took 5-6 weeks for the sucrose initially present in the culture medium to be exhausted. Similar rates of sucrose uptake by Arabidopsis cells cultivated in the light were measured. In the dark it was slightly higher (50-60 mg sucrose d^-1 ^g^-1 ^cell FW at 22°C). At 5°C, the sucrose initially added in NM was consumed within only 4-5 weeks. This indicates that the contribution of photosynthesis to sugar supply nearly compensated for the slightly higher growth rate of this cell strain. After exhaustion of the sucrose present in NM, the intracellular stores of carbohydrates are consumed and cell autophagy starts [15]. For this reason, it was necessary to subculture cells grown at 5°C at least every 4-5 weeks.

**Table 2:** Sucrose-uptake by sycamore cells incubated in Pi-supplied and Pi-deprived NM at 22°C and 5°C

	Pi-supplied NM		Pi- starved NM	
Temperature	22°C	5°C	22°C	5°C

Sucrose uptake (mg d^-1 ^g^-1^)	51 ± 5	9.7 ± 0.9	17 ± 1.5	3.4 ± 0.3

Cells were initially incubated for 5 d at 22°C in either Pi-supplied or Pi-free Lamport's NM. At day 5, incubation was pursued at 22°C or 5°C and sucrose-uptake expressed as mg d^-1 ^g^-1 ^cell FW was measured as indicated in "Material and Methods". Values are means ± SD (*n *= 5).

Subculturing cell cultures monthly might nevertheless still look as an excessive constraint. Thus, the question was to determine whether it was possible to further diminish the consumption of carbohydrates by cells whilst at the same time keeping cell suspensions physiologically safe. Since nearly one half of incorporated sugar is consumed by respiration and the other half by different metabolic pathways involved in cell growth [32], we stopped cell growth before lowering the temperature. For this we incubated cells in a Pi-free NM.

### Cell culture at 5°C in Pi-starved media and recovery

**Figure 5:**
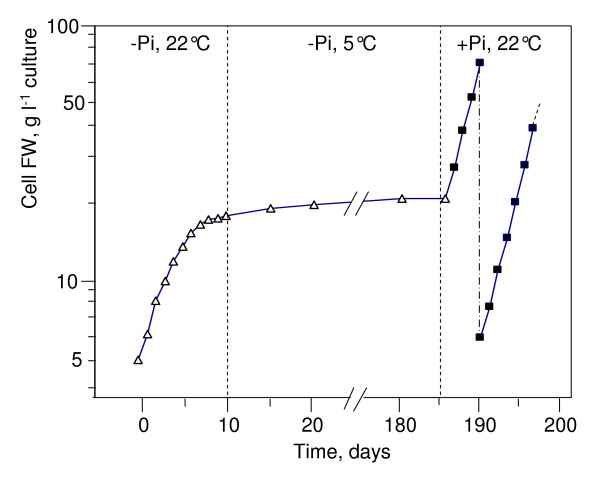
**Growth of sycamore cells under different culture conditions**. Growth of suspension-cultured sycamore cells was measured in a Pi-free nutrient medium at 22°C followed by cell preservation at 5°C, and recovery in a Pi-supplied medium at 22°C. Cells were cultivated as described in "Material and Methods". They originated from an exponentially growing cell suspension repeatedly subcultured in Pi-supplied NM before the growth plateau. At time zero, cells were incubated at 5.0 mg ml^-1 ^in Pi-free Lamport's NM at 22°C. After a short period of exponential growth, a growth plateau corresponding to a cell concentration of only 20 mg cell FW ml^-1 ^culture was attained due to Pi starvation. At that stage, the temperature was set to 5°C. Six months later preserved cells were incubated under standard culture conditions and subcultured (day 190). This experiment was repeated five times. In semi-logarithmic plot, standard deviation is of the order of the symbol size.

At 22°C, provided sucrose is added regularly to NM, it is possible to keep cell cultures alive in a Pi-free nutrient medium for several months. After a few days, the cell concentrations of Pi, phosphorylated intermediates of glycolysis, and nucleotides dramatically decrease [17,32], whereas the phospholipid/galactolipid ratio is reduced by ca. 70% [33,34]. Under these culture conditions, the growth of sycamore and Arabidopsis cells stops within one week (Figure 5 and Additional File 4). Afterwards, both the cell concentration and the rate of carbohydrate uptake from NM remained constant (Figure 4). Surprisingly, the cell respiration rate of Pi-deprived cells is only 30% lower (Table 3) than that of cells cultivated in standard NM (Table 1). In addition, the uncoupled respiration rates of P- deprived and Pi-supplied cells were similar, indicating that Pi-starvation did not modify the maximum potential activity of mitochondria. After the addition of Pi to NM, cell respiration recovered to normal values within minutes and cell ^31^P-NMR spectra showed a normal profile within a couple of hours [17].

**Table 3:** O_2_-uptake by sycamore and Arabidopsis cells incubated in Pi-free nutrient media at different temperatures

	sycamore		Arabidopsis	
	
Temperature		+ 2 μM FCCP		+ 2 μM FCCP
22°C	250 ± 25	510 ± 50	320 ± 30	690 ± 60

5°C	59 ± 6	135 ± 12	70 ± 7	190 ± 15

5°C→22°C	370 ± 30	530 ± 50	470 ± 40	720 ± 60

Sycamore and Arabidopsis cells were initially incubated for 5 d at 22°C in their respective NM devoid of Pi in order to stop cell growth. At day 5, incubation was pursued at 22°C and 5°C in the same nutrient media. O_2_-uptake by Pi-deficient cells was measured after 5 d incubation at 22°C and 6 months incubation at 5°C. In addition, the O_2_-uptake by cells incubated for 6 months at 5°C was measured at 22°C in standard Pi-supplied media after a 10-min recovery (5°C→22°C). All cells were incubated in the dark. O_2_-uptake rates measured as indicated in "Material and Methods" are expressed as nmol O_2 _consumed min^-1 ^g^-1 ^cell FW. Values are means ± SD (*n *= 5). FCCP, cyanide *p*-trifluoromethoxyphenylhydrazone.

When the incubation temperature of Pi-deprived cells was dropped to 5°C, a fivefold decrease of cell respiration was observed (Table 3), as mentioned above in the case of Pi-supplied cells, and the sucrose uptake from the culture medium diminished accordingly (see Figure 4 for sycamore cells). The mean sucrose uptake by sycamore cells was close to 3.4 mg d^-1 ^g^-1 ^cell FW (Table 2). This suggests, since their culture medium initially contains 20 g l^-1 ^sucrose, that it should be possible to keep alive 20 g of cells incubated at 5°C in one litre of a Pi-free NM culture over ca 10 months without renewing the culture medium.

**Figure 6:**
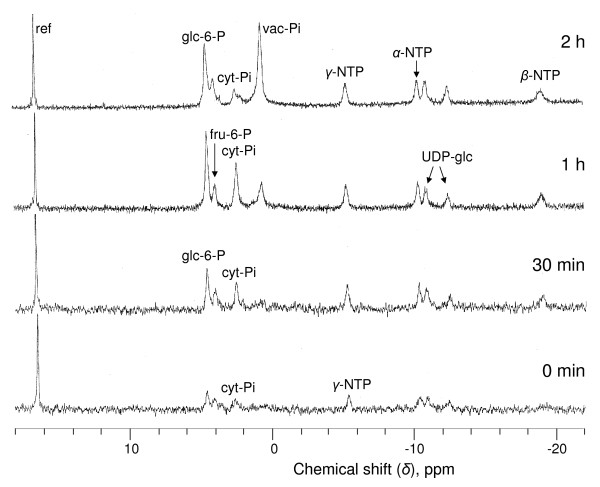
***In vivo *proton-decoupled ^31^P-NMR spectra showing the recovery of sycamore cells preserved over 6 months**. Cells were preserved over 6 months at 5°C in a Pi-free nutrient medium. They were then placed in the perfusion system dedicated to *in vivo *NMR analyses as described in "Material and Methods". At time zero, 100 μM Pi was added to NM and the temperature was set to 22°C. Spectra are the result of 1500 transients with a 0.6-s repetition time (15 min). Peak assignments are as follows: ref, reference (methylenediphosphonate) used to measure chemical shifts and for quantification; glc-6-P, glucose 6-phosphate; fru-6-P, fructose 6-P; cyt-Pi, cytoplasmic-Pi; vac-Pi, vacuolar Pi; NTP, nucleoside triphosphate; UDP-glc, uridine-5'-diphosphate-*α*-D-glucose.

After adding Pi to NMs and returning the temperature to 22°C, the cell respiration of cells stored during 6 months at 5°C in Pi free medium returned to standard values within minutes (Table 3). This suggests that cell suspensions remained physiologically homogenous after months of preservation at low temperature in Pi-free NMs. In particular, no appreciable cell death was measured. Accordingly, Figures 6 and Additional File 5 show examples of sycamore and Arabidopsis cell metabolite profile recovery after 6 months of incubation at 5°C in Pi-free NMs. At time zero, the concentration of cell's soluble P-compounds was close to the threshold for *in vivo *^31^P-NMR detection of ca 20 nmol. Thirty minutes after the addition of phosphate in the perfusing NMs, cytoplasmic Pi (cyt-Pi), and NTP recovered standard values, rapidly followed by glucose 6-P and UDP-glucose, and one hour later by vacuolar Pi. After two hours, the *in vivo *^31^P-NMR profiles of sycamore and Arabidopsis cells were close to those of standard cells. In fact, the time course recovery of the metabolite profile of Pi-starved cells incubated over 6 months at 5°C resembles that observed by Pratt et al. [17] for cells incubated 5 days in Pi-free NMs at 22°C. The analysis of *in vivo *spectra also indicated that the cytoplasmic pH of both types of cells, as measured from the chemical shift of cyt-Pi as soon as it was unambiguously identified (30 min), remained close to 7.4. This suggests that the regulation of the cytoplasmic pH was maintained by Pi-starved cells incubated at low temperature, as is the case at 22°C [32]. *In vivo *^13^C-NMR cell profiles of Pi-starved cells cultivated at 5°C were comparable to those of corresponding Pi-supplied cells. Similarly, a full recovery was observed within a few hours: hexoses were metabolised as well as accumulated amino acids.

Finally, the growth rates of cells incubated at low temperature in Pi-free NM rapidly recovered to standard values after the return to standard culture conditions. In particular, no appreciable delay was noticed (Figure 5) and cells can be further normally subcultured. This confirms that the preservation of culture cells at low temperature in a Pi-free medium not permitting cell growth, and without the addition of growth inhibitors, did not generate long-lasting physiological and metabolic changes. Importantly, *in vitro *and *in vivo *^13^C- and ^31^P-NMR analyses showed that the metabolic changes observed at low temperature in the absence of Pi in NM were rapidly reversed following the return to standard cell culture conditions. In addition, the absence of lag phase during the recovery of full cell respiration and growth rates suggests that the cell population remains physiologically homogenous during the preservation period.

The protocol for preserving plant cells without subculture over several months is described step-by-step in Additional file 6.

## Conclusion

The incubation of sycamore and Arabidopsis cells in a Pi-free nutrient medium at 5°C allowed the cell lines to stay alive for several months. This was due to the arrest of cell growth resulting from Pi starvation, which was initiated 10 days before lowering the temperature, and to the large decrease of cell metabolic activity at low temperature as indicated by the drop of respiration. Importantly, no cell death was observed and cells recovered a normal physiological and biochemical activity without a long delay or lag period.

To summarize, the procedure of cell preservation described in this paper opens the possibility of storing plant cells lines for several months whilst retaining their capacity to restart growing homogenously and without delay. It can be performed easily and routinely, without requiring any specific equipment, freezing procedure, or addition of growth inhibitors. This method for the preservation of suspension cultured cell lines, with a medium renewal every 6 months only, is now routinely used in our laboratory.

## Material and methods

### Plant material and growth conditions

Sycamore (*Acer pseudoplatanus *L.) and Arabidopsis (*Arabidopsis thaliana *L.) cells were respectively grown in Lamport [2] and Murashige and Skoog [35] nutrient media as described by Bligny and Leguay [36]. Both types of cells were aerated on orbital shakers monitored at 120 rpm in either a cell culture room at 22°C, or in a cold room at 5°C. Arabidopsis cells received continuous illumination delivering 100 μmol m^-2 ^s^-1 ^photosynthetic photon flux density (PPFD) whatever the temperature. Nutrient media contained 20 g l^-1 ^sucrose as carbohydrate source. At 22°C, cell suspensions were subcultured each 7 days, i.e. before sucrose present in NM was exhausted, by adding 40 ml of old cell culture to 200 ml of fresh NM in 800-ml flasks in order to obtain an initial cell concentration of nearly 5-10 mg FW ml^-1^. At 5°C cells were subcultured when 90% of the initially added sucrose was consumed.

### Metabolomic analyses using NMR

Analyses were performed either *in vitro *from perchloric acid (PCA) cell extracts or *in vivo *using freshly harvested cells. Extracts were prepared from 10-g (wet wt) cells quickly filtered, washed with pure water at 0°C, and thrown into liquid nitrogen. Frozen samples with 0.7 ml of 70% (v/v) PCA were ground to a fine powder with a mortar and pestle at liquid nitrogen temperature. The frozen powder was then thawed at 0°C and the resulting thick suspension was centrifuged at 15,000 *g *for 10 min at 0°C to remove particulate matter. The supernatant was neutralised to pH 5.0 with 2 M KHCO_3 _to precipitate PCA as KClO_4_, centrifuged at 15,000 *g *for 5 min and lyophilised. The freeze-dried material was dissolved in 2.0 ml water containing 10% ^2^H_2_O for further NMR adjustment and 1 μM sodium azide to avoid fermentation when unfrozen, and it was stored at -20°C.

*In vitro *NMR analyses were performed on a Bruker AMX 400 wide bore spectrometer (Bruker Instruments, Inc., Billerica, MA, USA) equipped with a 10-mm multinuclear-probe. The probe was tuned at 100.6 and 162.0 MHz for ^13^C- and ^31^P-NMR, respectively. The deuterium resonance of ^2^H_2_O was used as a lock signal. Spectra were recorded at 295 K. ^13^C-NMR spectra were the result of 3600 transients with a 6-s repetition time (6 h) recorded with 90° pulses (11 μs), a 20 kHz spectral width, and a Waltz-16 ^1^H decoupling sequence with 2.5 W and 0.5 W during acquisition time and delay, respectively. Free induction decays were collected as 32,000 data points, zero-filled to 64,000 and processed with a 0.2-Hz exponential line broadening. ^31^P-NMR spectra were the result of 1000 transients with a 3.6-s repetition time (1 h) recorded with 70° pulses (15 μs), a 8.2 kHz spectral width, and a Waltz-16 ^1^H decoupling sequence with 1 W during acquisition and 0.5 W during delay, respectively. Free induction decays were collected as 16,000 data points, zero-filled to 32,000 and processed with a 0.2-Hz exponential line broadening.

For ^13^C-NMR analyses, 4 μmol 1,2-cyclohexylenedinitrilotetraacetic acid (CDTA) was added to the PCA extract to chelate Mn^2+^, 150 μmol of maleate was added for calibration, and the pH was adjusted to 7.4. Spectra were referenced to the -CH = CH- peak of maleate positioned at 131.4 ppm. For ^31^P-NMR analyses, all divalent cations were chelated by the addition of sufficient amounts of CDTA, 2 μmol methylphosphonate was added for calibration, and the samples were buffered by addition of 150 μmol Hepes at pH 7.4. Spectra were referenced to the peak of methylphosphonate positioned at 22.67 ppm. The identification of the peaks of resonance was done by comparing the spectra of standard solutions of known compounds at pH 7.4 with that of the PCA extracts. The definitive assignments were made after running a series of spectra of the extracts spiked with authentic compounds, at different pHs to separate potentially overlapping peaks [18]. To accurately quantify compounds identified on spectra, the intensities of their different resonance peaks were referred to those of the reference compounds added to samples before grinding. Twenty seconds recycling time was used to obtain fully relaxed spectra. The integration function of the spectrometer was utilized to compare the intensity of resonance peaks.

*In vivo *NMR analyses were performed on the same spectrometer equipped with a 25-mm multinuclear-probe. The deuterium resonance of ^2^H_2_O was used as a lock signal. Cells (10 g FW) were placed in a 25-mm NMR tube and oxygenated as described by Aubert et al. [18] with a perfusion flux of 20 ml min^-1 ^sufficient for a perfect oxygenation of all cells at 22°C. The 4 l of perfusion NM contained the macro-nutrients (sucrose, KNO_3_, NH_4_NO_3_, KCl, Ca[NO_3_]_2_, and MgSO_4_) normally present in 200 ml of Lamport's or Murashige and Skoog's media, according to cell strains, which is sufficient for the growth of 10 g of cells over several days and limits decoupling-related temperature elevation at the level of analyzed cells. To further improve the signal-to-noise ratio, micro-nutrients, and in particular Mn^2+^, were not added to NM. The temperature of the perfusing NM was adjusted in a thermoregulated water bath outside the magnet.

^13^C-NMR spectra were acquired by accumulating 900 scans recorded with 90° pulses (70 μs) at 5.6-s intervals, a 20.7 kHz spectral width, and a Waltz-16 ^1^H decoupling sequence with 4 W and 0.5 W during acquisition time and delay, respectively. Free induction decays were collected as 16,000 data points, zero-filled to 32,000, and processed with a 2-Hz exponential line broadening. Spectra were referenced to hexamethyldisiloxane contained in a capillary inserted inside the central outlet perfusion tube at 2.7 ppm. ^31^P-NMR spectra were acquired as described by Pratt et al. [17]. Spectra were referenced to methylenediphosphonate (MDP, pH 8.9) contained in the same capillary, at 17.38 ppm.

The identification of the resonance peaks was performed by comparing the *in vivo *spectra of perfused cells to those of PCA extracts prepared from these cells and adjusted at different pHs, as described above. *In vivo *quantification was performed by comparing the spectra of analyzed cells with those of the extracts prepared from the same amount of cells, and using the MDP reference. The concentration of metabolites in the cytoplasm and in the vacuole were calculated as indicated in Pratt et al. [17]. Cytoplasmic and vacuolar pH (cyt- and vac-pH) was estimated from the chemical shift of the pools of Pi (cyt- and vac-Pi) present in these two compartments as described by Gout et al. [21].

### Other analytical methods

The cell samples FW and the growth of cell suspensions were measured as described by Bligny and Leguay [36]. The oxygen uptake by cells was measured at 5°C and 22°C in their respective cell culture media. O_2_-uptake was monitored polarographically using a Clark-type oxygen-electrode (Hansatech Ltd King's Lynn, UK). 50 mg cell (FW) was stirred in the 1 ml measurement chamber filled with NM. The O_2 _concentration in the air-saturated NM was taken as 250 μM at 22°C and 360 μM at 5°C according to Truesdale and Downing [37]. Uncoupled respiration was measured after adding 2 μM cyanide *p*-trifluoromethoxyphenylhydrazone (FCCP). For photosynthesis measurements, Arabidopsis cells were illuminated with 500 μmol m^-2 ^s^-1 ^PPFD.

The sucrose present in NM was measured as described by Bergmeyer [38], using invertase, hexokinase, and glucose 6-P dehydrogenase, and by ^13^C-NMR.

Neutral red was be used as a vital stain to detect the presence of dead cells in cultures.

When means ± SD are given, the statistical Student's *t*-test was applied to the data with *P *values ≤ 0.05.

## List of abbreviations

Pi: inorganic phosphate; PCA: perchloric acid; NM: nutrient medium; NMR: nuclear magnetic resonance.

## Competing interests

The authors declare that they have no competing interests.

## Authors' contributions

RB and CR conceived the study and drafted the manuscript. A-M B and EG carried out the analysis and contributed to draft the manuscript. All authors approved the final manuscript.

## Supplementary Material

Additional file 1 **Growth of suspension-cultured lightened Arabidopsis cells at 22°C (a) and 5°C (b)**. Legend as in Figure 1. Click here for file

Additional file 2 **Proton-decoupled ^13^C-NMR spectra of perchloric acid extracts of Arabidopsis cells grown in the light at 22°C (a) and 5°C (b)**. Legend as in Figure 2; fum, fumarate; Asp, aspartate; Glu, glutamate; Gln, glutamine. Click here for file

Additional file 3 **Proton-decoupled ^31^P-NMR spectra of perchloric acid extracts of Arabidopsis cells grown in the light at 22°C (a) and 5°C (b)**. Legend as in Figure 3; GPG, glycerophosphoglycerol; spectra are the result of 250 transients. Click here for file

Additional file 4 **Growth of suspension-cultured lightened Arabidopsis cells in a Pi-free nutrient medium at 22°C followed by cell preservation at 5°C, and recovery in a Pi-supplied medium at 22°C**. Legend as in Figure 5. Click here for file

Additional file 5 ***In vivo *proton-decoupled ^31^P-NMR spectra of Arabidopsis cells**. The recovery of preserved cell was followed *in vivo *after the return to standard perfusion conditions (Pi-supplied NM, 22°C) as indicated in the legend of Figure 6. Click here for file

Additional file 6 **Step-by-step description of the protocol for preserving plant cells without subculture over several months**. Click here for file
