# Supplementary figures and images for: A simple and efficient method for the long-term preservation of plant cell suspension cultures
Source: Plant Methods. 2012 Jan 30;8:4. doi: 10.1186/1746-4811-8-4 (PMC3284881; doi:10.1186/1746-4811-8-4)

## Additional file 1

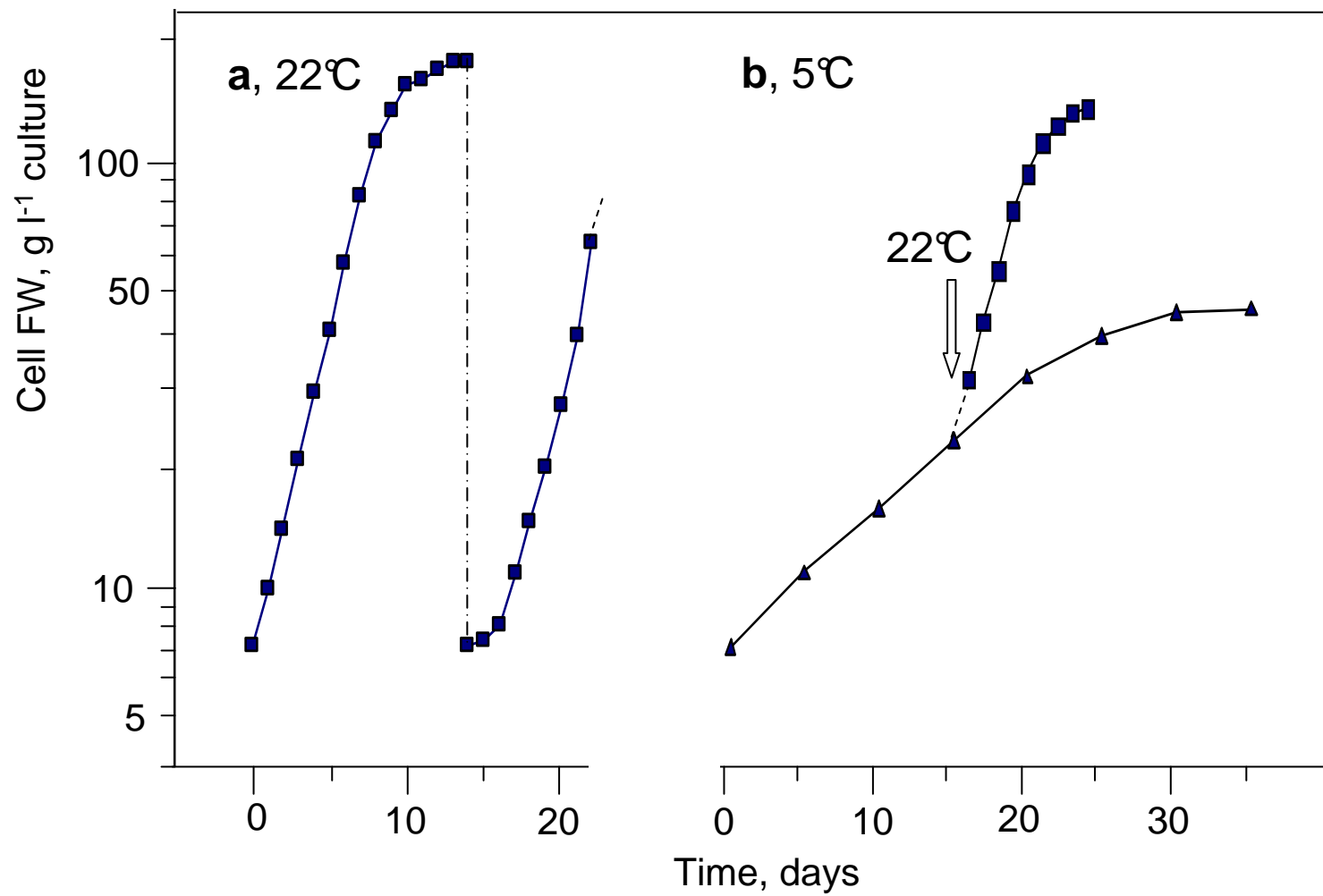

Supplement: Additional file 1 — Growth of suspension-cultured lightened Arabidopsis cells at 22°C (a) and 5°C (b). Legend as in Figure 1. [file 1746-4811-8-4-S1.PDF]

Additional file 2

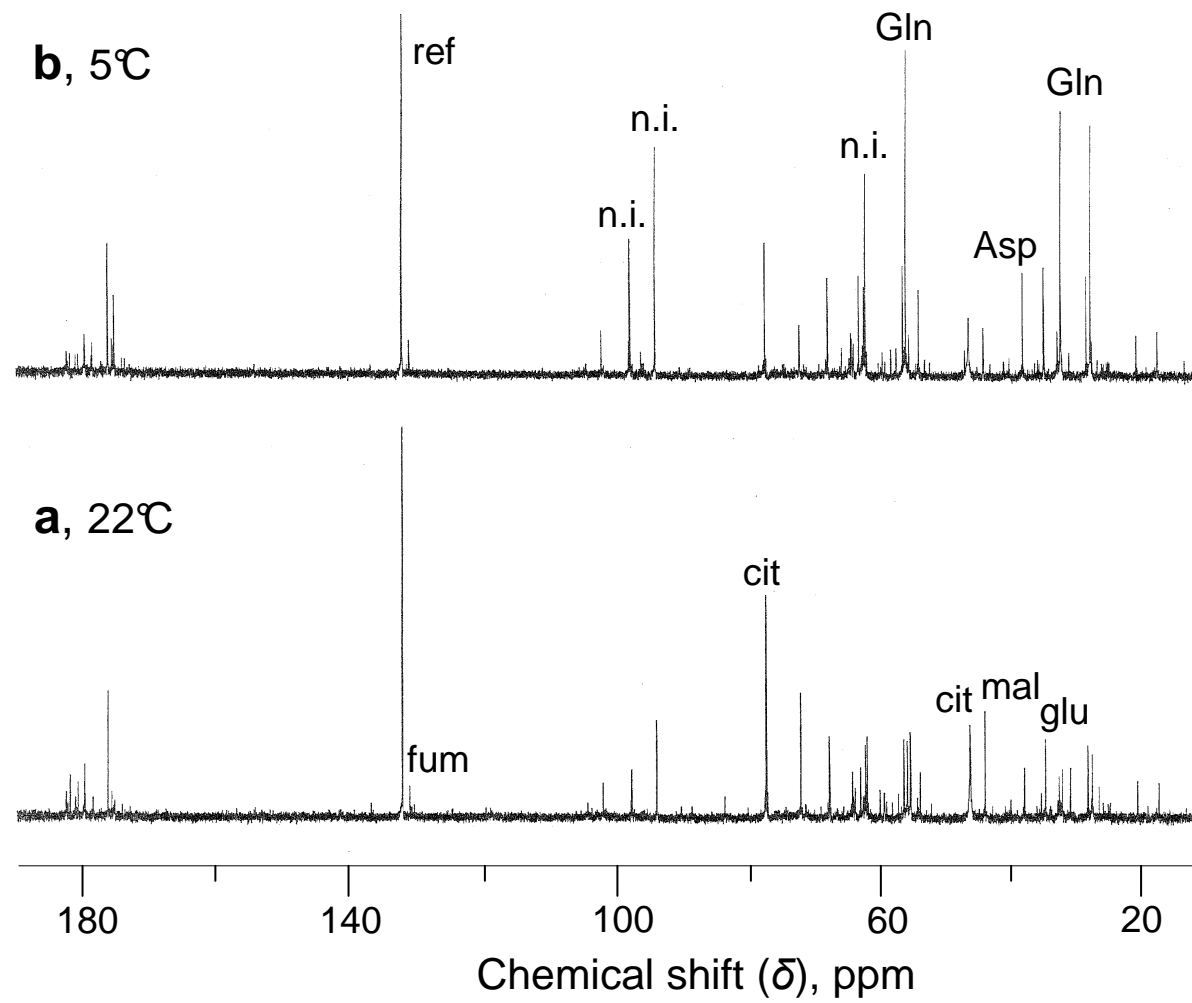

Supplement: Additional file 2 — Proton-decoupled 13C-NMR spectra of perchloric acid extracts of Arabidopsis cells grown in the light at 22°C (a) and 5°C (b). Legend as in Figure 2; fum, fumarate; Asp, aspartate; Glu, glutamate; Gln, glutamine. [file 1746-4811-8-4-S2.PDF]

Additional file 3

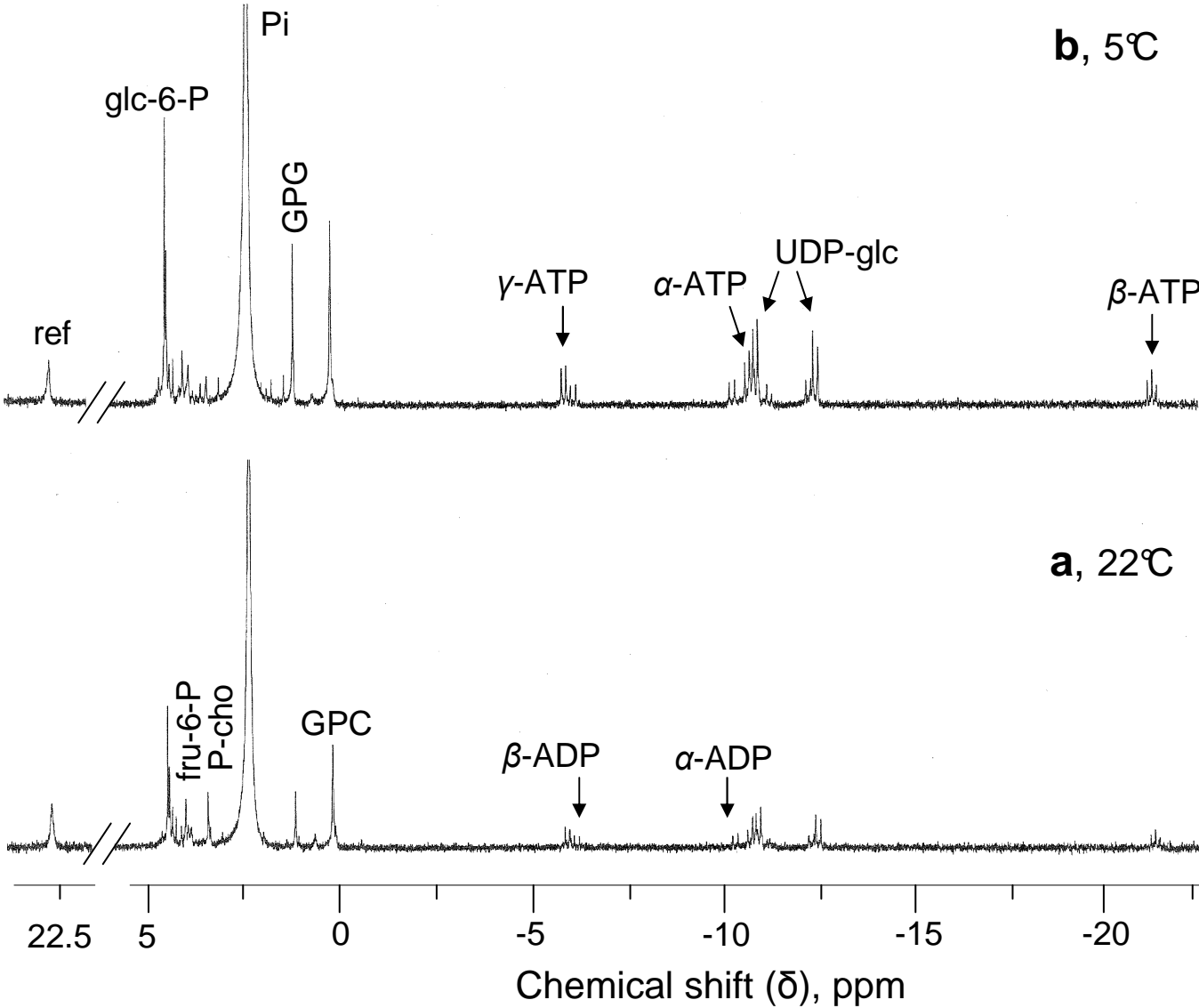

Supplement: Additional file 3 — Proton-decoupled 31P-NMR spectra of perchloric acid extracts of Arabidopsis cells grown in the light at 22°C (a) and 5°C (b). Legend as in Figure 3; GPG, glycerophosphoglycerol; spectra are the result of 250 transients. [file 1746-4811-8-4-S3.PDF]

## Additional file 4

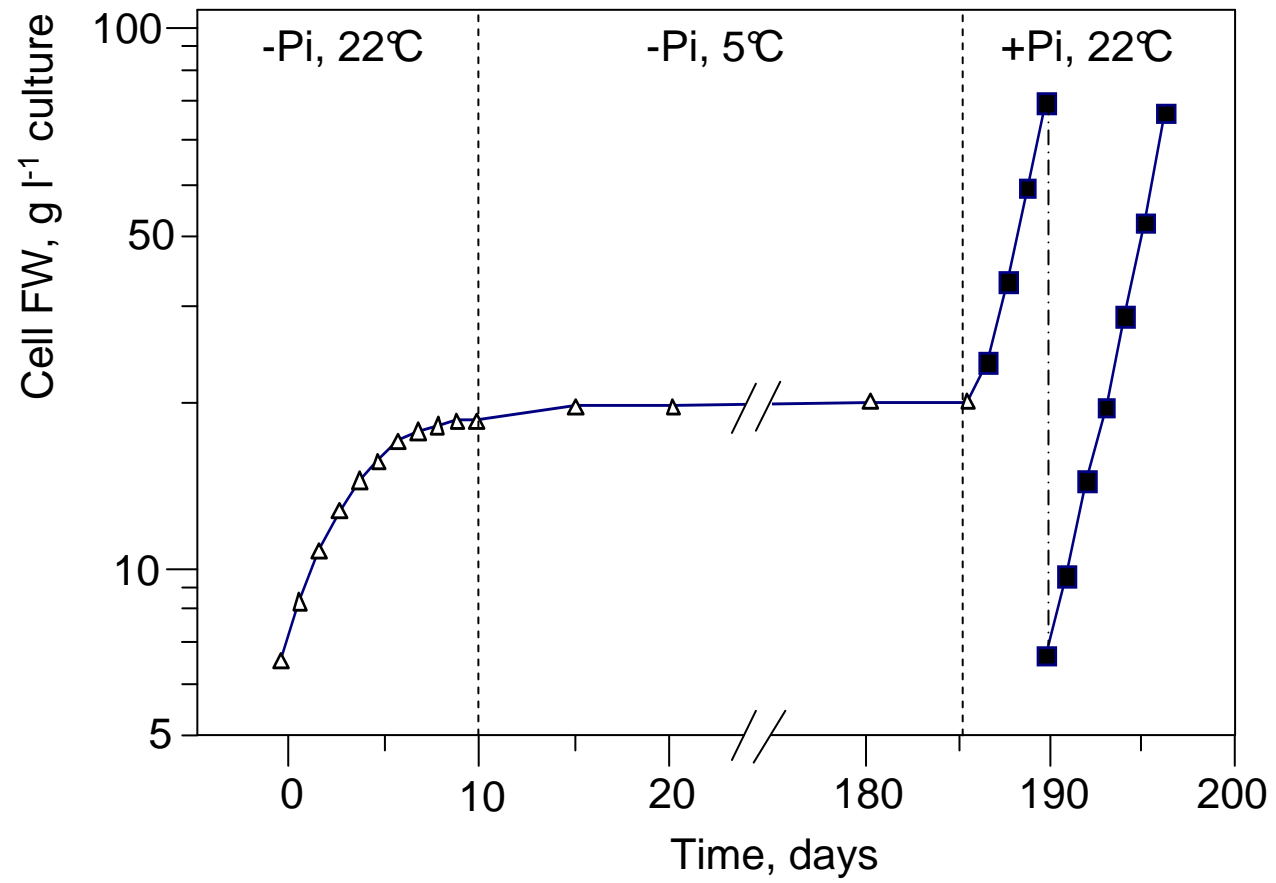

Supplement: Additional file 4 — Growth of suspension-cultured lightened Arabidopsis cells in a Pi-free nutrient medium at 22°C followed by cell preservation at 5°C, and recovery in a Pi-supplied medium at 22°C. Legend as in Figure 5. [file 1746-4811-8-4-S4.PDF]

# Additional file 5

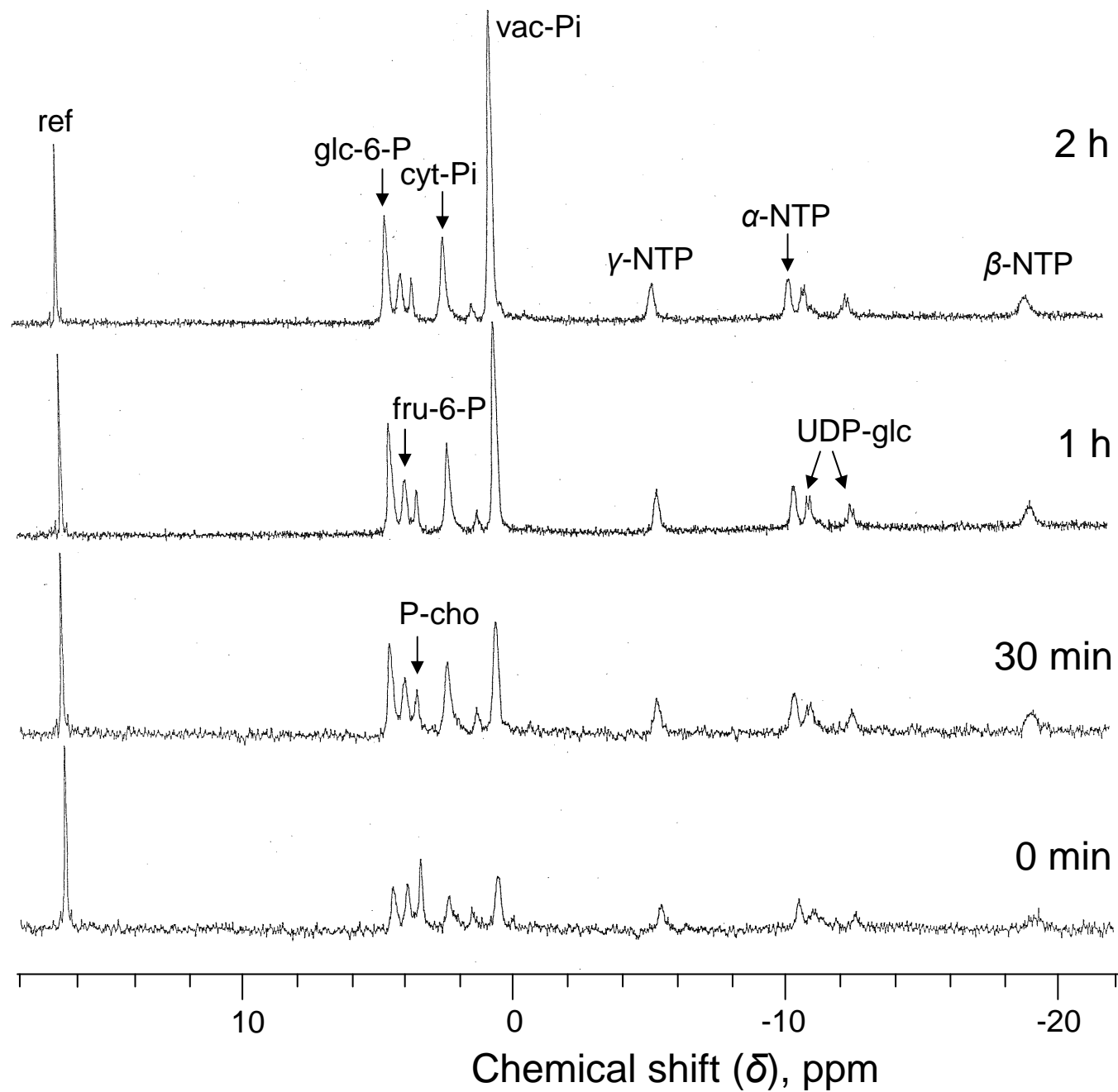

Supplement: Additional file 5 — In vivo proton-decoupled 31P-NMR spectra of Arabidopsis cells. The recovery of preserved cell was followed in vivo after the return to standard perfusion conditions (Pi-supplied NM, 22°C) as indicated in the legend of Figure 6. [file 1746-4811-8-4-S5.PDF]
