# Supplementary material for: A simple and efficient method for the long-term preservation of plant cell suspension cultures
Source: Plant Methods. 2012 Jan 30;8:4. doi: 10.1186/1746-4811-8-4 (PMC3284881; doi:10.1186/1746-4811-8-4)
Supplement: Additional file 6 — Step-by-step description of the protocol for preserving plant cells without subculture over several months. [file 1746-4811-8-4-S6.PDF]

Step-by-step description of the protocol for preserving plant cells without subculture over several months

1) **Standard cell culture**

Cells are routinely cultivated in suspension in 800 ml glass balloons containing 200 ml of nutrient media (see specific composition below). They are aerated on orbital shakers (Innova 2300, New Brunswick Scientific, Enfield, CT, USA) monitored at 120 rpm. Chlorophyllous cells (here Arabidopsis) receive a continuous illumination of 100 µmol m^{-2} s^{-1} photosynthetic photon flux density. The temperature of the growth room is maintained at 22°C. Cell suspensions are subcultured weekly by adding 20 ml of old cell culture to 200 ml of fresh NM in order to obtain an initial cell concentration of 5 mg FW ml^{-1}.

2) **Cell preservation in a Pi-free culture medium at low temperature**

Cell aliquots are transferred in their respective nutrient medium devoid of phosphate (*i.e.* without KH_2PO_4 and/or Na_2HPO_4), and incubated at 22°C for 10 days in order to let cell metabolize all the intracellular Pi stores, leading to the arrest of cell growth within about 7 days; the initial cell concentration, 5-7 mg FW ml^{-1}, reaches 20 mg FW ml^{-1} after 7 days of incubation in a Pi-free nutrient medium.

At day 10, Pi-free nutrient media are renewed and cell cultures balloons are placed in a cold room at 5°C, on orbital shakers monitored at 120 rpm as described for standard cultures. These Pi-starved cells can be kept at 5°C in Pi-free NM at a cell concentration of 20 mg cell FW per ml of culture over several months.

3) **Restarting culture growth**

To restart culture growth, cells are simply transferred in their respective normal, Pi-supplied, NM, and culture balloons are installed in the growth room at 22°C on the orbital shakers monitored at 120 rpm. Cultures restart growing exponentially without delay and cells are ready to be subcultured five days later.

Composition of nutrient media for sycamore and Arabidopsis cell culture:

	Sycamore	Arabidopsis
Macronutrients (mM)		
NH_4NO_3	-	20.6
KNO_3	22.0	18.8
$CaCl_2$	1.4	3.0
$MgSO_4$	1.5	1.5
KH_2PO_4	3.6	1.2
Na_2HPO_4	0.3	-
KCl	0.09	-
$Ca(NO_3)_2$	1.2	-
Micronutrients (µM)		
KI	4.5	5.0
H_3BO_3	24	100
$MnSO_4$	19	100
$ZnSO_4$	5.2	30
Na_2MoO_4	0.5	1.0
$CuSO_4$	0.2	0.1
$COCl_2$	0.1	0.1
$FeSO_4$ and Na_2EDTA	10	10
Organic compounds (µM)		
Inositol	-	100
Nicotinic acid	-	0.5
Pyridoxine · HCl	-	0.5
Thiamin · HCl	1.0	0.1
IAA	-	1.3
Kinetin	-	0.1
2,4-D	1.0	-
Sucrose	20,000	30,000
pH	5.5	5.7
